# Secular trend analysis of antibiotic utilisation in China’s hospitals 2011–2018, a retrospective analysis of procurement data

**DOI:** 10.1186/s13756-020-00709-6

**Published:** 2020-04-15

**Authors:** Haishaerjiang Wushouer, Yue Zhou, Xi Zhang, Mengyuan Fu, Daiming Fan, Luwen Shi, Xiaodong Guan

**Affiliations:** 1 Center for Strategic Studies, Chinese Academy of Engineering, No.2 Bingjiaokou HuTong, Xicheng District, Beijing, 100088 China; 2 School of Medicine, Tsinghua University, No.30 Shuangqing Road, Haidian District, Beijing, 100084 China; 3 International Research Center for Medicinal Administration (IRCMA), Peking University, No.38 Xueyuan Road, Haidian District, Beijing, 100191 China; 4 Department of Pharmacy Administration and Clinical Pharmacy, School of Pharmaceutical Sciences, Peking University, No.38 Xueyuan Road, Haidian District, Beijing, 100191 China; 5 State Key Laboratory of Cancer Biology, National Clinical Research Center for Digestive Diseases, Xijing Hospital of Digestive Diseases, Air Force Military Medical University, No. 15 Changlexi Road, Xincheng District, Xi’an, 710032 China; 6 Department of Population Medicine, Harvard Medical School and Harvard Pilgrim Health Care Institute, 133 Brookline Avenue, Boston, MA 02215 USA

**Keywords:** Trend, Antibiotic, Utilisation, Hospitals, China

## Abstract

**Background:**

This study was aimed to explore the secular trends of antibiotic utilisation in China’s hospitals over an 8-year period.

**Methods:**

We retrospectively analysed aggregated monthly antibiotic procurement data of 586 hospitals from 28 provinces in China from January 2011 to December 2018. Information including generic name, procurement amount, dosage form, strength, the route of administration, and geographical data were collected. Population weighted antibiotic utilisation were expressed in DDD per 1000 inhabitants per day (DID). WHO’s ‘Access, Watch, Reserve’ categorization was also adopted to analyse antibiotic utilisation.

**Results:**

Between 2011 and 2018, total antibiotic utlisation in China’s hospitals increased by 39.6% (from 4.8 DID in 2010 to 6.7 DID in 2018). Antibiotic utilisation was stable or had moderately decreased in 13 provinces, while in the other 15 provinces they had substantially increased. Cephalosporins were the most consumed antibiotics, accounting for 26.9% of the total antibiotic utilisation (1.8 DID/6.7 DID). In 2018, antibiotics in the Access category comprised 19.4% of the total utilisation (1.3 DID/6.7 DID), where antibiotics in the Watch category comprised the largest proportion of 71.6% (4.8 DID/6.7 DID). Population-weighted antibiotic utlisation was greater in secondary hospitals than in tertiary hospitals (7.3 DID vs 6.6 DID). The utilisation of oral forms was almost two times the amount of parenteral forms in secondary hospitals, whereas in tertiary hospitals the amounts were about the same.

**Conclusions:**

Despite efforts have been made towards restricting antibiotic use by the Chinese government, antibiotic utilisation demonstrated an upward trend after the medical reform. The increase of last-resort antibiotics raises serious concern for public health. Current patterns of antibiotic utilisation demonstrated that gaps are existed towards the global target set up by the WHO. To better facilitate proper antibiotic use, more efforts are needed to explore the appropriateness of antibiotic use at the individual level.

## Background

Selection pressure from antibiotic use is considered to be one of the most important drivers of antimicrobial resistance (AMR), which is a growing public health threat of broad concern to the world [1]. At the G20 Hangzhou Summit in 2016, AMR was one of the main topics and was included in the final communiqué. At the 71st UN General Assembly, AMR was the fourth health-related topic that was discussed in UN General Assembly in history. It has been estimated that more than two million lives would be at risk, and up to 3.5 billion US dollars will be spent annually on average due to AMR in Europe, North America and Australia by 2050 if no action is taken [2]. According to the theory of choice, there is an expected net benefit of antibiotics use to the individual. Therefore, protecting the efficacy of antibacterial agents and confining AMR should be a common goal of every country. China is one of the largest consumers of antibiotics in the world and is also under the threat of AMR [3].

To address this issue, China has taken many measures to strengthen antimicrobials management such as the designation of antibiotics as prescription drugs in 2003, and the introduction of guidance for clinical use of antibiotics in 2004 [4, 5]. National surveillance networks for both antibiotic use and resistance were established in 2005, along with the introduction of a national formulary in 2008 [6, 7]. However, due to the inefficient implementation and the absence of supervision and inspection, these policies and strategies did not fully achieve the expected results [3]. The overuse of antimicrobials has remained a serious public health challenge. Since the 2009 health system reform, the Chinese government has been committed to tackling the inappropriate use of antibiotics by enhancing antimicrobial stewardship. World Health Organization (WHO) called for urgent and concerted action to slow down the emergence and spread of drug resistance, with the slogan of “no action today, no cure tomorrow” in 2011. Echoing the global governance endeavors of the WHO, the National Health and Family Planning Commission (NHFPC) launched a three-year Special Antimicrobial Stewardship Campaign (SAC) nationwide to strengthen the management of clinical use antimicrobials in healthcare settings [8]. The SAC was legislated then as a ministerial decree in 2012 [9]. In addition, structured antimicrobial formulary restriction management has been established, which categorized antimicrobials into three classes (non-restricted, restricted and highly-restricted), with different prescription privileges to different levels of physicians. In 2016, a national action plan to confine AMR was released responding to WHO’s call for the “Global Action Plan on Antimicrobial Resistance” [10]. Moreover, to respond to the emerging carbapenem-resistant bacteria, the NHFPC made specific and more restrictive requirements for the clinical use of carbapenems and tigecycline in 2018 [11].

We have studied the short-term trends of antibiotic utilisation by analysing the antibiotic procurement data (how much antibiotics were bought by the hospitals) in China’s tertiary hospitals before [12]. However, the long-term change in antibiotic use, especially after nearly a decade since health system reform, was yet to be explored. Hence, as an extension of our previous study, with new indicators and covering secondary hospitals, this study was designed to explore the secular trends of antibiotic utilisation by analysing procurement data over an 8-year period.

## Methods

### Study design

Aggregated monthly antibiotic procurement records of 586 hospitals from 28 provinces were retrospectively analysed for the study period from January 2011 to December 2018.

### Data source

All the data was obtained from China Medicine Economic Information (CMEI), an observational database containing information of drug procurement records in medical institutions from 28 provinces (out of 34) across the country (Qinghai, Tibet, Hainan, Hongkong, Macau, and Taiwan excluded). The database covers more than 1000 city-level public hospitals, including outpatient and inpatient, across mainland China. The procurement records of participating hospitals, which were all public hospitals, account for approximately 40% of total drug procurement at city-level public hospitals in China [13]. Since antibiotics in Chinese public hospitals were acquired through a centralised procurement mechanism, all the acquired antibiotics were included in the database. Procurement records in CMEI were provided as acquisition data of the participating hospitals. For simplicity, we call these “antibiotic utilisation” when we referred to hospitals. Hospitals were sampled hierarchically based on geographical and socio-economic factors. We selected the hospitals on the basis that they each had full records of antibiotic procurement records during the entire study period. Based on this criterion, the selected 452 tertiary hospitals accounted for 21.9% of the total tertiary hospitals and the selected 134 secondary hospitals accounted for 2.3% of the total secondary hospitals in the study regions (Table 1).

**Table 1:** Distribution of sample hospitals

Region ^a^	Tertiary ^b^	Secondary ^c^
Eastern	256/982 (26.1)	73/1991 (3.7)
Middle	132/602 (21.9)	36/2132 (1.4)
Western	64/484 (13.2)	25/1763 (1.7)
Total	452/2068 (21.9)	134/5886 (2.3)

^a^: Classification of the regions was obtained from the China Health Statistics Yearbook. Eastern region: Beijing, Tianjin, Hebei, Liaoning, Shanghai, Jiangsu, Zhejiang, Shandong, and Guangdong; Middle region: Shanxi, Jilin, Heilongjiang, Anhui, Jiangxi, Henan, Hubei, and Hunan; Western region: Inner Mongolia, Chongqing, Guangxi, Sichuan, Guizhou, Yunnan, Shaanxi, Gansu, Ningxia, and Xinjiang

^b^: Percentage in parenthesis was calculated by dividing the number of sampled tertiary hospitals by the total number of tertiary hospitals in the region

^c^: Percentage in parenthesis was calculated by dividing the number of sampled secondary hospitals by the total number of secondary hospitals in the region

### Data collection and management

We extracted monthly antibiotic procurement data from the CMEI database. Information including the generic name, procurement amount, dosage form, strength, the route of administration, and geographical data were collected. Hospital names were concealed to protect confidentiality.

Procurement data were categorized according to Anatomical Therapeutic and Chemical (ATC) classification J01 (i.e. antibacterial for systemic use) expressed in defined daily dose (DDD) as a measurement unit, following the recommendation of the WHO Collaborating Center for Drug Statistic Methodology [14]. The DDD of the drugs which could not be coded in the ATC system was calculated as the recommended daily amount for each study medication based on the dosage regimen recommended in the manufacturers’ instructions, as approved by China Food and Drug Administration. A total of 186 unique chemical substance names were identified in single or combination antibiotics. These antibiotics were aggregated into 32 ATC-4 classes and then into 9 ATC-3 groups. Data were managed and analysed in Microsoft Excel 2013 and STATA 14.0 (StataCorp LLC, Texas, USA).

### Data analysis

To make the procurement data consistent with the international standards, the data were converted into DDD per 1000 inhabitants per day (DID) at the level of the active substance. Based on the following two assumptions, eq. 1 was adopted to calculate the weighted population as a proxy for the population our sample hospitals had covered. The first assumption was that there was no significant difference in the distribution of the sample hospitals across the provinces; Second, there was no significant difference in the distribution of the population which was covered by the sample hospitals across the provinces. To avoid bias in calculating inhabitants, the inhabitants’ coverage was calculated together for outpatient and inpatient, instead of separate calculation as we did before [12]. The inhabitants’ coverage for secondary and tertiary hospitals were calculated separately.

1 $$ {Y}_i={\sum}_{i=1}^{28}{P}_i\times \frac{n_i}{N_i}\times \frac{m_i}{M_i} $$

Y_i_: Coverage inhabitants in a given year;

P_i_: Total population in a given year in province i;

n_i_: Number of sample hospitals in province i;

N_i_: Number of total hospitals in province i;

m_i_: Number of inpatients and outpatients in sample hospitals in province i;

M^i^: Number of inpatients and outpatients in all hospitals in province i.

In addition to ATC classification, we adopted ‘Access, Watch, Reserve’ (AWaRe) categorization established by WHO as part of the update of the WHO Model List of Essential Medicines in 2017 to analyse the antibiotic utilisation [15].

To derive a comparable metric of antibiotic utilisation across time, we calculated the compound annual growth rate (CAGR) of antibiotic utilisation [3].

2 $$ \mathrm{CAGR}={\left(\frac{C_{2018}}{C_{2011}}\ \right)}^{\frac{1}{4}}-1 $$

*C*_2018_: Total antibiotic utilisation for the year 2018 (expressed in DID).

*C*_2011_: Total antibiotic utilisation for the year 2011 (expressed in DID).

All the relevant census data for calculating inhabitants were collected from China Health Statistics Yearbook and China Statistics Year Book [16]. Linear regression analysis was adopted to determine the trends in antibiotic use with time. A difference with *p* < 0.05 was considered to indicate statistical significance.

## Results

### Total antibiotic utilisation in China’s hospitals from 2011 to 2018

Between 2011 and 2018, total antibiotic utilisation in China’s hospitals increased by 39.6% (from 4.8 DID in 2011 to 6.7 DID in 2018). The CAGR of the total antibiotic utilisation was 4.3%. In 2018, antibiotics in the Access category comprised 19.4% of total utilisation (1.3 DID/6.7 DID), whereas antibiotics in the Watch category represented the largest proportion of all – 71.6% (4.8 DID/6.7 DID). The percentage of antibiotics in the Access, Watch and Reserve categories was stable during the study period.

**Fig. 1:**
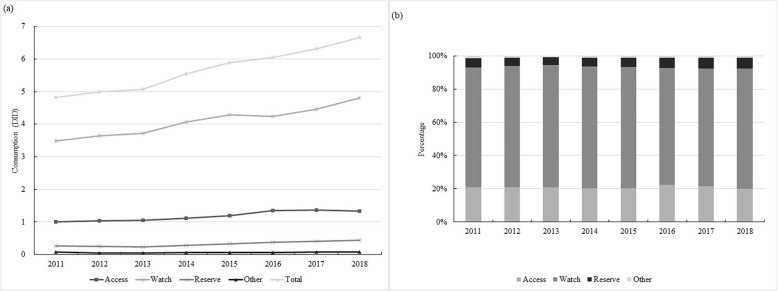
Antibiotic utilisation under the WHO AWaRe category in China’s hospitals, 2011–2018. **a** Expressed in DID. **b** Expressed in cumulative percentage

Cephalosporins were the most consumed antibiotics, accounting for 26.9% of the total antibiotic utilisation in 2018 (1.8 DID/6.7 DID), followed by combinations of penicillins with 22.4% (1.5 DID/ 6.7 DID) and fluoroquinolones with 10.4% (0.7 DID/6.7 DID). The largest absolute increase between 2011 and 2018 was observed for combinations of penicillins (0.6 DID), cephalosporins (0.3 DID), and fluoroquinolones (0.2 DID). The most important relative increase from 2011 was observed for carbapenems (233.3%, from 0.03 DID to 0.1 DID), tetracyclines (200.0%, from 0.1 DID to 0.3 DID), other antibacterials (fosfomycin, daptomycin, spectinomycin, linezolid, methenamine) (50.0%, from 0.4 DID to 0.6 DID), combinations of penicillins (50.0%, from 1.0 DID to 1.5 DID) and penicillins with extended spectrum (50.0%, from 0.2 DID to 0.3 DID). (Fig. 1, and Additional file 1).

**Fig. 2:**
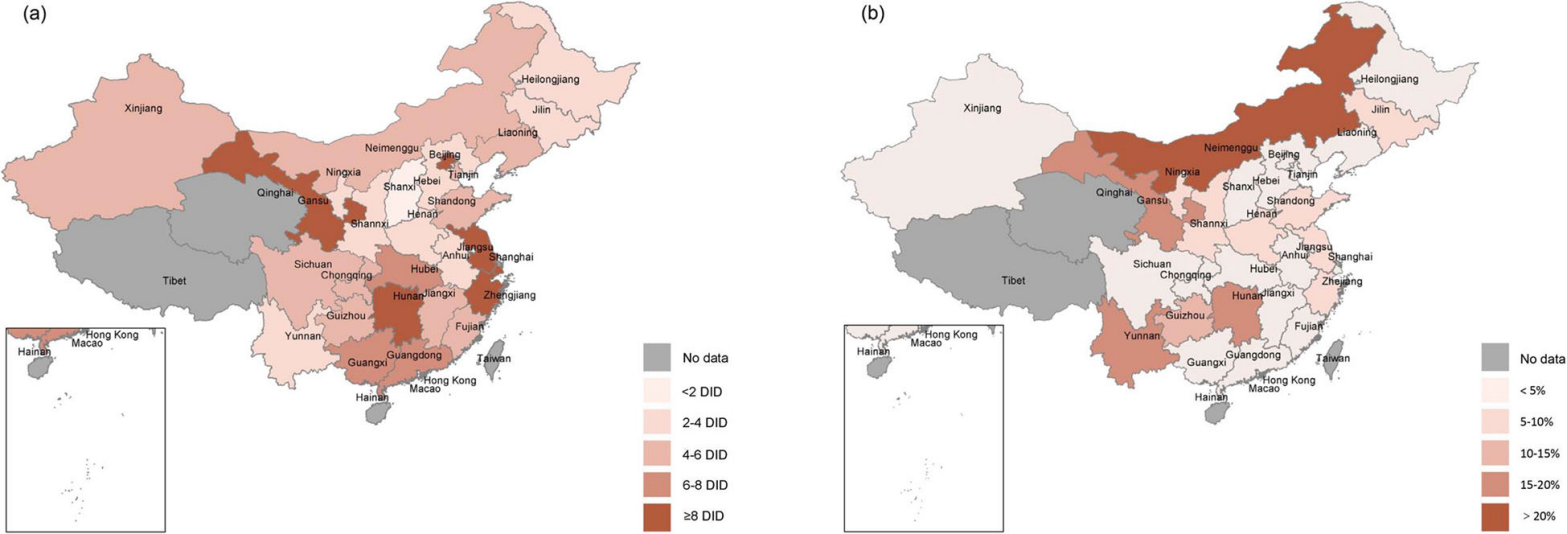
Antibiotic utilisation in China’s hospitals in 2018. **a** Expressed in DID; **b** Expressed in compound annual growth rates of antibiotic utilisation between 2011 and 2018

Beijing, Jiangsu, and Shanghai were the top three largest consumers of antibiotics in 2018, with 18.3 DID, 16.7 DID, and 11.6 DID, respectively. Antibiotic utlisation was stable or had moderately decreased in half of the country (13 provinces), while in the other half (15 provinces) they had substantially increased between 2011 and 2018. Most of the increases were observed in underdeveloped regions, such as Inner Mongolia, Yunnan, Gansu, and Guizhou. Eastern coastal regions consumed more antibiotics compared with central and western regions (Fig. 2).

### Antibiotic utlisation in secondary and tertiary hospitals

**Fig. 3:**
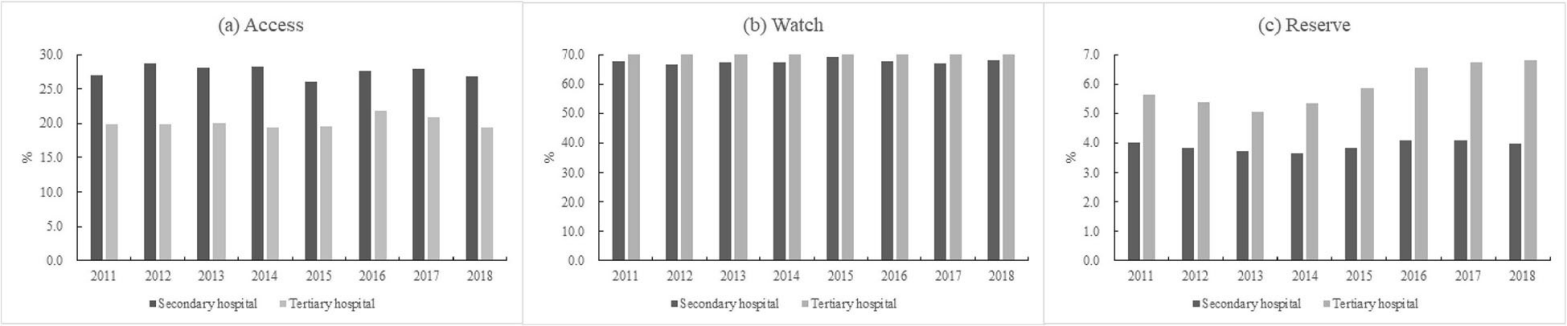
Antibiotic utlisation under the WHO AWaRe category in secondary and tertiary hospitals, 2011–2018

In 2018, total antibiotic utlisation in secondary and tertiary hospitals were 7.3 DID and 6.6 DID, respectively. Antibiotics in the Access category comprised 27.4% (2.0 DID/7.3 DID) and 19.7% (1.3 DID/6.6 DID) of total amount in secondary and tertiary hospitals, respectively, whereas antibiotics in the Watch category had the largest proportion of 68.5% (5.0 DID/7.3 DID) and 72.7% (4.8 DID/ 6.6 DID), respectively. Antibiotics in the Reserve category in tertiary hospitals increased from 4.1% (0.3 DID/ 7.3 DID) to 7.6% (0.5 DID/ 6.6 DID) during the study period (Fig. 3).

**Fig. 4:**
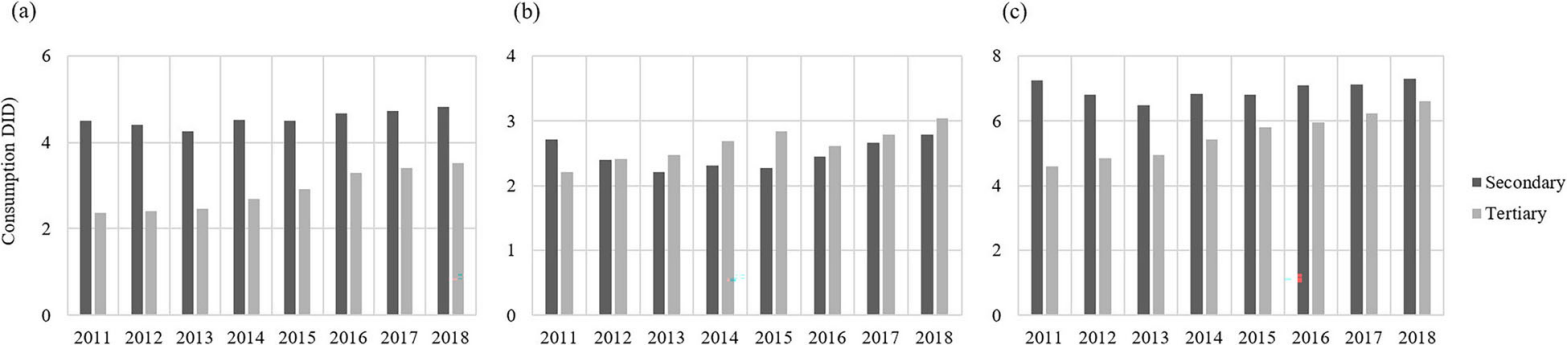
Utilisation of oral and parenteral antibiotics in secondary and tertiary hospitals between 2011 and 2018. **a** Oral antibiotics; **b** Parenteral antibiotics; **c** Total antibiotic utilisation

Antibiotic utilisation in secondary hospitals fell by 6.9% (7.3 DID to 6.8 DID) from 2011 to 2015 and rose by 7.4% (6.8 DID to 7.3 DID) from 2015 to 2018. In tertiary hospitals, antibiotic utlisation increased each year, and a 43.5% increase was observed between 2011 and 2018 (4.6 DID to 6.6 DID). The utilisation of oral forms was almost two times the amount of parenteral forms in secondary hospitals, whereas in tertiary hospitals the amounts were about the same (Fig. 4).

**Fig. 5:**
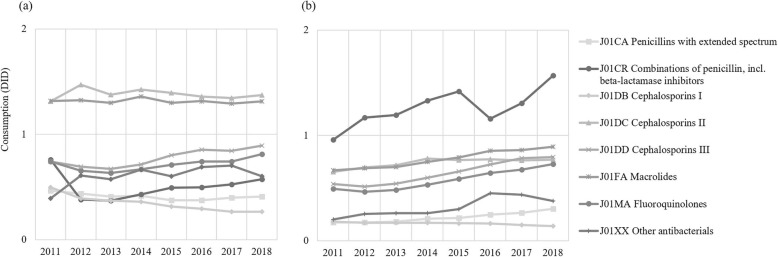
Major classes of antibiotics utilised in secondary and tertiary hospitals between 2011 and 2018. **a** Secondary hospitals; **b** Tertiary hospitals

As Fig. 5 showed, the three most commonly utilised antibiotics in secondary hospitals were second-generation cephalosporins, macrolides, and third-generation cephalosporins, whereas the combinations of penicillins, which increased by 63.5% from 0.96 DID to 1.57 DID between 2011 and 2018, replaced second-generation cephalosporins as one of the top three in tertiary hospitals in 2018 (Fig. 3).

## Discussion

In this study, we estimate secular trends of antibiotic utilisation in China’s secondary and tertiary hospitals over an 8-year period, using procurement data from a national sampling database and the accepted ATC/DDD methodology. This allows us to analyse the antibiotic utilisation in China’s hospital setting and benchmark it internationally, which would be informing for healthcare providers, decision-makers, as well as the public.

In the nearly past two decades, the Chinese government has attempted to confine AMR by a series of policies and measures including limiting antibiotic use. The evolution of the policy management has been well documented [9]. According to the National Health Commission, the outpatient antibiotic prescription proportion in Chinese hospitals (secondary and tertiary hospitals) has continuously declined from 16.2% in 2011 to 7.7% in 2017. Similarly, the inpatient antibiotic prescription proportion has also declined from 55.2 to 38.0% [17]. In addition, studies were conducted on the impact of antibiotic use in secondary and tertiary hospitals, analysing the effect of various drug management programs on promoting the proper use of antibiotics. It should be noted that the implementation of the zero mark-up policy cut off the economic incentives between the volume of prescriptions and the hospital revenue [12, 18, 19]. However, our study showed that antibiotic utilisation in China’s hospitals increased significantly (38.2% increase between 2011 and 2018). Although the developed regions with larger populations consumed more antibiotics, significant increases were observed in underdeveloped regions despite having smaller populations (4 out of the top 5 provinces with the highest increase in antibiotic utilisation were underdeveloped). While it can be hard to explain the discrepancy with other studies that describe a decrease in antibiotic use [20–23], we found evidence that is consistent with the increasing tendency, when considering changes in the pharmaceutical market and inpatient volume. China’s anti-infection pharmaceutical market continuously increased from 2011 to 2017 with varying growth rates. Meanwhile, the inpatient volume at China’s tertiary hospitals nearly tripled between 2010 to 2017 according to China Health Statistics Yearbook [16]. This indicated that the demand for antibiotic treatment was growing. What’s more, although the proportion of antibiotic prescriptions was decreasing, the median antibiotic usage intensity in 177 core members of the Centre for Antibacterial Surveillance was slightly increasing from 2014 to 2016 from 47.21 to 47.65 DDDs/100 patient days. In addition, the methodology in our study was different from the one used in the national report. The difference stems from the latter report extracting data from the Centre for Antibacterial Surveillance, which is a national surveillance network for collecting antibiotic prescription data across more than 1300 hospitals nationwide. This might indicate that the composition of the sample hospitals between CMEI database and national surveillance network was different. However, due to the lack of access to the list of sample hospitals, a broader study with more hospitals covered is needed to verify the difference.

When compared with European countries, our results showed that antibiotic utilisation in China’s hospitals was more than three times than the average level (mean) of antibiotic consumption in the hospital setting in 24 EU/EEA countries (2.0 DID in 2017, ranged from 0.9 to 3.1 DID) [24]. One more worrying fact was that the antibiotic utlisation in China’s hospitals increased by a CAGR of 4.3% while the same metric, taken on an average annual basis in EU/EEA countries’ hospital settings was 1.0% [24]. However, these results need to be interpreted with caution, because in EU/EEA countries outpatient antibiotic use accounted for 90–94% of the total (considering countries that provided separate data in ESAC) [24, 25], whilst the majority of antibiotics were consumed in a hospital setting in China. This might explain why the antibiotic utilisation in China’s hospitals was lower than that of antibiotics consumed in the community setting in EU/EEA countries (6.3 DID vs 21.8 DID in 2017) [24]. AWaRe categories are proposed by WHO in the context of a comprehensive review of the optimal antibiotic choices for many common infectious syndromes in adults and children. WHO set up a global target of having the proportion of Access antibiotics be greater than 60% of all antibiotics prescribed to reduce AMR [26]. Our study showed that the Access category proportion in the sample hospitals was only 19.4%, which was lower than the proportion in most countries that collected this data [26]. The massive use of second- and third-generation cephalosporins, macrolides, as well as combinations of penicillins contributed to the high proportion of the Watch category. Measuring antibiotic utilisation by quantifying the antibiotic use in each of the AWaRe categories allows some inference about the overall quality of antibiotic use between countries. The combination of both absolute and relative utilisation by category allows simple benchmarking (e.g. an overuse of Watch antibiotics can become immediately apparent and a reduction in Watch antibiotics can be identified as a target for antibiotic stewardship interventions) and assessment of trends over time (to evaluate the impact of interventions) [2].

When looking at the pattern of antibiotic utilisation, we found that cephalosporins were continuously the most consumed antibiotics in China’s hospitals, followed by combinations of penicillins, which were often defined as extended-spectrum antibiotics, and quinolones. This pattern was similar to the previous studies conducted in other regions of China [18, 19], as well as national surveillance data [17]. Unlike in China, more penicillins were prescribed in Europe and the United States [27–29]. This might partly be attributed to the different settings that antibiotics were consumed: mostly inpatient in China compared to an ambulatory setting in Europe and the US. Cephalosporins were recommended by the national guidance for the majority of the perioperative prophylaxis settings in China [30]. Although quinolones were recommended by US FDA to those who have no other treatment options [31], Chinese physicians may prefer quinolones and cephalosporins more than penicillins due to the time-consuming skin allergy testing requirements for penicillins prior to administration [32]. Although the utilisation of carbapenems, a class of last-resort antibiotics, were similar to EU/EEA countries (0.10 DID vs 0.06 DID in 2017, country range: 0.02–0.17), the significant increase of carbapenems utilisation still cannot be ignored (0.03 DID in 2011 to 0.10 DID in 2018) [24]. Because carbapenems are categorized as highly restricted antibiotics by the National Health Commission and require pre-authorization before use in China, this increase could be partly due to the rise in extended-spectrum β-lactamase-producing Gram-negative bacteria, which has been identified in epidemiological surveillance studies [17, 33]. Alongside the increase of carbapenem utilisation, the resistance rate of carbapenem-resistant Enterobacteriaceae has also been reported [34].

Population-weighted antibiotic utilisation was greater in secondary hospitals than in tertiary hospitals. As more severe patients were admitted in tertiary hospitals compared with secondary hospitals, we speculated that more potential inappropriate use of antibiotics might exist in lower level hospitals. Studies found that diseases, such as diarrheal illness, colds, pharyngitis, acute bronchitis, were likely to be prescribed antibiotics in rural and underdeveloped regions [35–37], although most of these illnesses are viral instead of bacterial. This is consistent with our results that most of the increases in antibiotic utilisation were observed in underdeveloped regions. However, further study on the level of individual antibiotic usage, such as a manual medical record review, is needed to confirm our speculation.

The findings of this study are subject to several limitations. First, the hospitals in the database were on a voluntary basis instead of mandatory participation, especially the proportion of secondary hospitals in the study was relatively low, therefore could bring selection bias. Besides, the sample hospitals in our study were all public hospitals and the sample size was relatively small, however, since the number of patient visits in public hospitals was more than 85% of total patient visits in China [16], and the CMEI database covered about 40% of total drug procurement at city-level public hospitals, therefore it still made our study to be representative to a certain extent. Second, the population denominator used in the study was determined under certain conditions which may underestimate antibiotic utilisation as it cannot include cross-provincial patient flow. Third, since we cannot access bed day data and the size of the hospital, the estimation of the population covered by the sample hospitals might be biased. Finally, as the study analysed procurement data rather than clinical usage of antibiotics, we were unable to determine the appropriateness of antibiotic use at the individual level.

## Conclusions

Despite efforts have been made towards restricting antibiotic use in the past decade by the Chinese government, antibiotic utilisation demonstrated an upward trend after the medical reform. The increase of antibiotic utilisation, especially the increase of last-resort antibiotics raises serious concern for public health. Current patterns of antibiotic utilisation demonstrated that gaps are existed towards the global target set up by the WHO. To better facilitate proper antibiotic use, more efforts are needed to explore the appropriateness of antibiotic use at the individual level.

## Supplementary information

**Additional file 1: Table S1.** Antibiotic utilisation in China’s hospitals between 2011 and 2018.

## Data Availability

Aggregated annual procurement data used for analyses are provided as supporting information. The raw individual hospital level data used to generate these results cannot be shared publicly because of the associated legislation. Data can be made available by China Pharmacy Association (contact via tel. 86 10 65660788 or fax 86 10 65661656) for researchers who meet the criteria for access to confidential data and subject to a data access fee.

## Abbreviations

DDD: Defined daily dose
DID: DDD per 1000 inhabitants per day
AMR: Antimicrobial resistance
WHO: World Health Organization
NHFPC: National Health and Family Planning Commission
SAC: Special Antimicrobial Stewardship Campaign
CMEI: China Medicine Economic Information
ATC: Anatomical Therapeutic and Chemical
AwaRe: Access, Watch, Reserve
CAGR: Compound annual growth rate
