# Supplementary material for: Secular trend analysis of antibiotic utilisation in China’s hospitals 2011–2018, a retrospective analysis of procurement data
Source: Antimicrob Resist Infect Control. 2020 Apr 15;9:53. doi: 10.1186/s13756-020-00709-6 (PMC7160954; doi:10.1186/s13756-020-00709-6)
Supplement: Supplementary file 1 — Additional file 1: Table S1. Antibiotic utilisation in China’s hospitals between 2011 and 2018. [file 13756_2020_709_MOESM1_ESM.docx]

**Table S1**. Antibiotic utilisation in China’s hospitals between 2011 and 2018.

|  |  |  | **Consumption/DID** | | | | | | | |
| --- | --- | --- | --- | --- | --- | --- | --- | --- | --- | --- |
|  |  |  | **2011** | **2012** | **2013** | **2014** | **2015** | **2016** | **2017** | **2018** |
| **Secondary hospital** | |  |  |  |  |  |  |  |  |  |
|  | J01A |  |  |  |  |  |  |  |  |  |
|  |  | J01AA | 0.13 | 0.10 | 0.10 | 0.10 | 0.09 | 0.13 | 0.16 | 0.16 |
|  | J01B |  |  |  |  |  |  |  |  |  |
|  |  | J01BA | 0.00 | 0.00 | 0.00 | 0.00 | 0.00 | 0.00 | 0.00 | 0.00 |
|  | J01C |  |  |  |  |  |  |  |  |  |
|  |  | J01CA | 0.46 | 0.44 | 0.41 | 0.42 | 0.37 | 0.37 | 0.40 | 0.41 |
|  |  | J01CE | 0.25 | 0.22 | 0.18 | 0.18 | 0.19 | 0.28 | 0.31 | 0.35 |
|  |  | J01CF | 0.01 | 0.00 | 0.00 | 0.00 | 0.00 | 0.00 | 0.01 | 0.02 |
|  |  | J01CG | 0.00 | 0.00 | 0.00 | 0.00 | 0.00 | 0.00 | 0.00 | 0.00 |
|  |  | J01CR | 0.76 | 0.38 | 0.37 | 0.43 | 0.49 | 0.50 | 0.52 | 0.57 |
|  | J01D |  |  |  |  |  |  |  |  |  |
|  |  | J01DA | 0.05 | 0.02 | 0.02 | 0.02 | 0.02 | 0.01 | 0.01 | 0.01 |
|  |  | J01DB | 0.50 | 0.39 | 0.37 | 0.36 | 0.31 | 0.29 | 0.26 | 0.26 |
|  |  | J01DC | 1.31 | 1.47 | 1.38 | 1.42 | 1.39 | 1.36 | 1.34 | 1.37 |
|  |  | J01DD | 0.74 | 0.69 | 0.67 | 0.71 | 0.80 | 0.85 | 0.84 | 0.89 |
|  |  | J01DE | 0.02 | 0.02 | 0.02 | 0.02 | 0.02 | 0.02 | 0.02 | 0.02 |
|  |  | J01DF | 0.02 | 0.03 | 0.02 | 0.02 | 0.03 | 0.03 | 0.01 | 0.01 |
|  |  | J01DH | 0.01 | 0.02 | 0.02 | 0.02 | 0.03 | 0.04 | 0.04 | 0.05 |
|  |  | J01DI | 0.00 | 0.00 | 0.00 | 0.00 | 0.00 | 0.00 | 0.00 | 0.00 |
|  | J01E |  |  |  |  |  |  |  |  |  |
|  |  | J01EA | 0.00 | 0.00 | 0.00 | 0.00 | 0.00 | 0.00 | 0.00 | 0.00 |
|  |  | J01EB | 0.00 | 0.00 | 0.00 | 0.00 | 0.00 | 0.00 | 0.00 | 0.00 |
|  |  | J01EC | 0.01 | 0.01 | 0.01 | 0.01 | 0.01 | 0.01 | 0.01 | 0.01 |
|  |  | J01EE | 0.00 | 0.00 | 0.00 | 0.00 | 0.00 | 0.00 | 0.00 | 0.00 |
|  | J01F |  |  |  |  |  |  |  |  |  |
|  |  | J01FA | 1.32 | 1.32 | 1.30 | 1.36 | 1.30 | 1.31 | 1.29 | 1.31 |
|  |  | J01FF | 0.12 | 0.11 | 0.10 | 0.11 | 0.11 | 0.12 | 0.11 | 0.14 |
|  |  | J01FG | 0.02 | 0.00 | 0.00 | 0.00 | 0.00 | 0.00 | 0.00 | 0.00 |
|  | J01G |  |  |  |  |  |  |  |  |  |
|  |  | J01GA | 0.00 | 0.00 | 0.00 | 0.00 | 0.00 | 0.00 | 0.00 | 0.00 |
|  |  | J01GB | 0.13 | 0.11 | 0.10 | 0.11 | 0.11 | 0.10 | 0.08 | 0.07 |
|  | J01M |  |  |  |  |  |  |  |  |  |
|  |  | J01MA | 0.74 | 0.65 | 0.63 | 0.67 | 0.71 | 0.74 | 0.74 | 0.81 |
|  |  | J01MB | 0.00 | 0.00 | 0.00 | 0.00 | 0.00 | 0.00 | 0.00 | 0.00 |
|  | J01X |  |  |  |  |  |  |  |  |  |
|  |  | J01XA | 0.00 | 0.00 | 0.00 | 0.01 | 0.01 | 0.01 | 0.01 | 0.01 |
|  |  | J01XB | 0.00 | 0.00 | 0.00 | 0.00 | 0.00 | 0.00 | 0.00 | 0.00 |
|  |  | J01XC | 0.00 | 0.01 | 0.01 | 0.01 | 0.01 | 0.01 | 0.01 | 0.01 |
|  |  | J01XD | 0.20 | 0.18 | 0.17 | 0.19 | 0.18 | 0.19 | 0.19 | 0.18 |
|  |  | J01XE | 0.02 | 0.01 | 0.02 | 0.01 | 0.01 | 0.02 | 0.01 | 0.02 |
|  |  | J01XX | 0.39 | 0.61 | 0.57 | 0.66 | 0.60 | 0.69 | 0.70 | 0.60 |
| **Tertiary hospital** |  |  |  |  |  |  |  |  |  |  |
|  | J01A |  |  |  |  |  |  |  |  |  |
|  |  | J01AA | 0.12 | 0.12 | 0.12 | 0.14 | 0.16 | 0.20 | 0.22 | 0.25 |
|  | J01B |  |  |  |  |  |  |  |  |  |
|  |  | J01BA | 0.01 | 0.01 | 0.00 | 0.00 | 0.00 | 0.00 | 0.00 | 0.00 |
|  | J01C |  |  |  |  |  |  |  |  |  |
|  |  | J01CA | 0.18 | 0.18 | 0.18 | 0.21 | 0.22 | 0.25 | 0.27 | 0.30 |
|  |  | J01CE | 0.17 | 0.18 | 0.19 | 0.21 | 0.23 | 0.22 | 0.22 | 0.21 |
|  |  | J01CF | 0.01 | 0.01 | 0.01 | 0.01 | 0.01 | 0.01 | 0.01 | 0.01 |
|  |  | J01CG | 0.00 | 0.00 | 0.00 | 0.00 | 0.00 | 0.00 | 0.00 | 0.00 |
|  |  | J01CR | 0.96 | 1.17 | 1.19 | 1.33 | 1.42 | 1.16 | 1.31 | 1.57 |
|  | J01D |  |  |  |  |  |  |  |  |  |
|  |  | J01DA | 0.03 | 0.01 | 0.01 | 0.01 | 0.01 | 0.01 | 0.01 | 0.02 |
|  |  | J01DB | 0.18 | 0.17 | 0.17 | 0.17 | 0.17 | 0.16 | 0.15 | 0.14 |
|  |  | J01DC | 0.65 | 0.70 | 0.72 | 0.78 | 0.77 | 0.77 | 0.76 | 0.77 |
|  |  | J01DD | 0.54 | 0.51 | 0.54 | 0.60 | 0.66 | 0.73 | 0.78 | 0.79 |
|  |  | J01DE | 0.03 | 0.02 | 0.02 | 0.02 | 0.02 | 0.02 | 0.02 | 0.02 |
|  |  | J01DF | 0.03 | 0.02 | 0.01 | 0.01 | 0.01 | 0.01 | 0.01 | 0.01 |
|  |  | J01DH | 0.03 | 0.04 | 0.05 | 0.06 | 0.08 | 0.10 | 0.10 | 0.11 |
|  |  | J01DI | 0.00 | 0.00 | 0.00 | 0.00 | 0.01 | 0.01 | 0.01 | 0.01 |
|  | J01E |  |  |  |  |  |  |  |  |  |
|  |  | J01EA | 0.00 | 0.00 | 0.00 | 0.00 | 0.00 | 0.00 | 0.00 | 0.00 |
|  |  | J01EB | 0.00 | 0.00 | 0.00 | 0.00 | 0.00 | 0.00 | 0.00 | 0.00 |
|  |  | J01EC | 0.01 | 0.01 | 0.01 | 0.01 | 0.02 | 0.02 | 0.02 | 0.02 |
|  |  | J01EE | 0.00 | 0.00 | 0.00 | 0.00 | 0.00 | 0.00 | 0.00 | 0.00 |
|  | J01F |  |  |  |  |  |  |  |  |  |
|  |  | J01FA | 0.67 | 0.69 | 0.70 | 0.75 | 0.79 | 0.85 | 0.86 | 0.89 |
|  |  | J01FF | 0.03 | 0.04 | 0.04 | 0.04 | 0.04 | 0.04 | 0.05 | 0.06 |
|  |  | J01FG | 0.00 | 0.00 | 0.00 | 0.00 | 0.00 | 0.00 | 0.00 | 0.00 |
|  | J01G |  |  |  |  |  |  |  |  |  |
|  |  | J01GA | 0.01 | 0.01 | 0.01 | 0.01 | 0.00 | 0.00 | 0.00 | 0.00 |
|  |  | J01GB | 0.08 | 0.07 | 0.07 | 0.07 | 0.08 | 0.08 | 0.07 | 0.07 |
|  | J01M |  |  |  |  |  |  |  |  |  |
|  |  | J01MA | 0.49 | 0.46 | 0.48 | 0.53 | 0.59 | 0.64 | 0.68 | 0.73 |
|  |  | J01MB | 0.00 | 0.00 | 0.00 | 0.00 | 0.00 | 0.00 | 0.00 | 0.00 |
|  | J01X |  |  |  |  |  |  |  |  |  |
|  |  | J01XA | 0.01 | 0.01 | 0.01 | 0.02 | 0.02 | 0.02 | 0.02 | 0.02 |
|  |  | J01XB | 0.00 | 0.00 | 0.00 | 0.00 | 0.00 | 0.00 | 0.00 | 0.00 |
|  |  | J01XC | 0.01 | 0.01 | 0.01 | 0.02 | 0.02 | 0.02 | 0.02 | 0.02 |
|  |  | J01XD | 0.14 | 0.13 | 0.14 | 0.15 | 0.15 | 0.16 | 0.16 | 0.16 |
|  |  | J01XE | 0.01 | 0.00 | 0.01 | 0.01 | 0.03 | 0.02 | 0.02 | 0.02 |
|  |  | J01XX | 0.20 | 0.25 | 0.26 | 0.26 | 0.30 | 0.45 | 0.44 | 0.38 |
| **Total** |  |  |  |  |  |  |  |  |  |  |
|  | J01A |  |  |  |  |  |  |  |  |  |
|  |  | J01AA | 0.12 | 0.12 | 0.10 | 0.14 | 0.16 | 0.19 | 0.21 | 0.25 |
|  | J01B |  |  |  |  |  |  |  |  |  |
|  |  | J01BA | 0.01 | 0.00 | 0.00 | 0.00 | 0.00 | 0.00 | 0.00 | 0.00 |
|  | J01C |  |  |  |  |  |  |  |  |  |
|  |  | J01CA | 0.20 | 0.20 | 0.20 | 0.22 | 0.23 | 0.26 | 0.28 | 0.31 |
|  |  | J01CE | 0.17 | 0.18 | 0.19 | 0.21 | 0.23 | 0.22 | 0.23 | 0.22 |
|  |  | J01CF | 0.01 | 0.01 | 0.01 | 0.01 | 0.01 | 0.01 | 0.01 | 0.01 |
|  |  | J01CG | 0.00 | 0.00 | 0.00 | 0.00 | 0.00 | 0.00 | 0.00 | 0.00 |
|  |  | J01CR | 0.94 | 1.10 | 1.12 | 1.26 | 1.34 | 1.10 | 1.24 | 1.49 |
|  | J01D |  |  |  |  |  |  |  |  |  |
|  |  | J01DA | 0.03 | 0.01 | 0.01 | 0.01 | 0.01 | 0.01 | 0.01 | 0.02 |
|  |  | J01DB | 0.21 | 0.19 | 0.19 | 0.19 | 0.18 | 0.17 | 0.16 | 0.15 |
|  |  | J01DC | 0.71 | 0.76 | 0.77 | 0.83 | 0.82 | 0.82 | 0.81 | 0.82 |
|  |  | J01DD | 0.56 | 0.53 | 0.55 | 0.61 | 0.67 | 0.74 | 0.79 | 0.80 |
|  |  | J01DE | 0.03 | 0.02 | 0.02 | 0.02 | 0.02 | 0.02 | 0.02 | 0.02 |
|  |  | J01DF | 0.03 | 0.03 | 0.01 | 0.01 | 0.01 | 0.01 | 0.01 | 0.01 |
|  |  | J01DH | 0.03 | 0.04 | 0.04 | 0.06 | 0.08 | 0.09 | 0.10 | 0.10 |
|  |  | J01DI | 0.00 | 0.00 | 0.00 | 0.00 | 0.01 | 0.01 | 0.01 | 0.01 |
|  | J01E |  |  |  |  |  |  |  |  |  |
|  |  | J01EA | 0.00 | 0.00 | 0.00 | 0.00 | 0.00 | 0.00 | 0.00 | 0.00 |
|  |  | J01EB | 0.00 | 0.00 | 0.00 | 0.00 | 0.00 | 0.00 | 0.00 | 0.00 |
|  |  | J01EC | 0.01 | 0.01 | 0.01 | 0.01 | 0.02 | 0.02 | 0.02 | 0.02 |
|  |  | J01EE | 0.00 | 0.00 | 0.00 | 0.00 | 0.00 | 0.00 | 0.00 | 0.00 |
|  | J01F |  |  |  |  |  |  |  |  |  |
|  |  | J01FA | 0.72 | 0.74 | 0.75 | 0.80 | 0.83 | 0.89 | 0.90 | 0.93 |
|  |  | J01FF | 0.04 | 0.04 | 0.04 | 0.04 | 0.05 | 0.05 | 0.06 | 0.07 |
|  |  | J01FG | 0.00 | 0.00 | 0.00 | 0.00 | 0.00 | 0.00 | 0.00 | 0.00 |
|  | J01G |  |  |  |  |  |  |  |  |  |
|  |  | J01GA | 0.01 | 0.00 | 0.01 | 0.00 | 0.00 | 0.00 | 0.00 | 0.00 |
|  |  | J01GB | 0.08 | 0.07 | 0.07 | 0.08 | 0.08 | 0.08 | 0.08 | 0.07 |
|  | J01M |  |  |  |  |  |  |  |  |  |
|  |  | J01MA | 0.51 | 0.48 | 0.49 | 0.54 | 0.60 | 0.65 | 0.68 | 0.74 |
|  |  | J01MB | 0.00 | 0.00 | 0.00 | 0.00 | 0.00 | 0.00 | 0.00 | 0.00 |
|  | J01X |  |  |  |  |  |  |  |  |  |
|  |  | J01XA | 0.01 | 0.01 | 0.01 | 0.02 | 0.02 | 0.02 | 0.02 | 0.02 |
|  |  | J01XB | 0.00 | 0.00 | 0.00 | 0.00 | 0.00 | 0.00 | 0.00 | 0.00 |
|  |  | J01XC | 0.01 | 0.01 | 0.01 | 0.01 | 0.02 | 0.02 | 0.02 | 0.02 |
|  |  | J01XD | 0.15 | 0.14 | 0.14 | 0.15 | 0.16 | 0.16 | 0.16 | 0.16 |
|  |  | J01XE | 0.01 | 0.00 | 0.01 | 0.01 | 0.03 | 0.02 | 0.02 | 0.02 |
|  |  | J01XX | 0.22 | 0.28 | 0.29 | 0.29 | 0.33 | 0.47 | 0.46 | 0.40 |
